# CpG island methylation status and mutation analysis of the *RB1* gene essential promoter region and protein-binding pocket domain in nervous system tumours

**DOI:** 10.1038/sj.bjc.6600737

**Published:** 2003-01-28

**Authors:** P Gonzalez-Gomez, M J Bello, M E Alonso, D Arjona, J Lomas, J M de Campos, A Isla, J A Rey

**Affiliations:** 1 Departmento de C. Experimental, Laboratorio de Oncogenetica Molecular, Hospital Universitario La Paz, Madrid, Spain; 2 Departamento de Neurocirugía, Hospital del Rio Hortega, Valladolid, Spain; 3 Departamento de Neurocirugía, Hospital Universitario La Paz, Madrid, Spain

**Keywords:** *RB1* gene, CpG island, hypermethylation, mutation, nervous system tumours

## Abstract

A series of 136 nervous system tumours were studied to determine the methylation status of the CpG island contained within the promoter region of the *RB1* gene, as well as mutation analysis of the essential promoter region and exons 20–24 (and surrounding intronic regions) coding for the protein-binding pocket domain. Methylation of the *RB1* CpG island was detected in 26 samples corresponding to nine glioblastomas, three anaplastic astrocytomas, one mixed oligo-astrocytoma, one ependymoma, two medulloblastomas, two primary central nervous system lymphomas, two neurofibrosarcomas, and six brain metastasis from solid tumours. No inactivating mutations were found within the *RB1* promoter region, whereas one glioblastoma and one oligodendroglioma displayed similar sequence variations consisting of 12 and 8 base pair deletions at intron 21. These results suggest that *RB1* CpG island hypermethylation is a common epigenetic event that is associated with the development of malignant nervous system tumours.

The retinoblastoma susceptibility gene (*RB1*) is located on the long arm of chromosome 13 (at 13q14) and represents the classical example of a tumour suppressor gene. It spreads over 200 kb and encodes a nucleoprotein (pRB) that plays a key role in the cell cycle regulation complexes that govern the G1–S transition of cells, thus allowing mitosis and cell division. In late G1, pRB is phosphorylated by the cyclin D1/cyclin-dependent kinase 4/6 complex, producing the release of nuclear proteins and transcription factors (E2F family); this results in the progression of the cell cycle into the S phase. On the other hand, the hypophosphorylated form of pRB induces a G1 cell cycle arrest (Friend *et al*, 1986; Lee *et al*, 1987; Buchkovich *et al*, 1989). The activity of the cyclin D1/cyclin-dependent kinases complex is controlled by inhibiting proteins such as p16 (CDKN2A), and the loss of *p16* or *RB1* function would result in a deregulated cell proliferation (Medema *et al*, 1995).

Loss of *RB1* function has been described in a variety of tumour types, and significant association has been observed between loss of heterozygosity (LOH) of *RB1* intragenic markers and the absence of pRB expression. LOH at the *RB1* locus has been found in 25–45% of glioblastomas and in about 25% of anaplastic astrocytomas (AA), as well as in bladder carcinomas, and malignant neuroendocrine lung carcinomas (Ishikawa *et al*, 1991; Hogg *et al*, 1993; Xu *et al*, 1993; Gouyer *et al*, 1994; Henson *et al*, 1994; Ichimura *et al*, 1996). Sequencing analysis of all 27 exons of the *RB1* gene in those neoplasms with LOH at the *RB1* locus showed a low rate (5–12%) of inactivating mutations, suggesting the location of another tumour suppressor gene at 13q14 or the existence of another *RB1* silencing mechanism, such as promoter hypermethylation (Ishikawa *et al*, 1991; Ichimura *et al*, 1996; Tsuzuki *et al*, 1996; Ueki *et al*, 1996). The loss of pRB expression may also be the result of mutation or microdeletion of the *RB1* promoter region, as described in hereditary retinoblastoma and prostate cancer (Bookstein *et al*, 1990a; Sakai *et al*, 1991). Alternatively, these molecular alterations have also been described involving the *RB1* protein-binding pocket domain, producing structurally and functionally altered pRB proteins (Yandell *et al*, 1989; Hensel *et al*, 1990; Mori *et al*, 1990). Loss of pRB expression with no association to LOH of the *RB1* intragenic markers has been described in breast carcinomas, prostate cancer, and pituitary adenomas, reinforcing the existence of alternative *RB1* gene-inactivating mechanisms (Borg *et al*, 1992; Cooney *et al*, 1996; Simpson *et al*, 1999).

Methylation of gene regulatory elements is an epigenetic change representing an alternative to genetic alteration for gene inactivation. Methylation of CpG islands located within a promoter element is generally associated with delayed replication and inhibition of transcription initiation (Baylin *et al*, 1998; Delgado *et al*, 1998; Jones and Laird, 1999). The *RB1* gene could be inactivated by a combination of genetic and epigenetic alterations of two alleles; in fact, the *RB1* gene harbours a CpG island that encompasses the essential promoter region, which is unmethylated during development (Jones, 1996). Experimental data show that *in vitro* methylation of the *RB1* promoter region reduces pRB expression (Ohtani-Fujita *et al*, 1993) and unilateral retinoblastoma frequently shows loss of pRB expression associated with aberrant methylation of CpG island within the *RB1* promoter region (Stirzaker *et al*, 1997).

To determine the mechanisms participating in an inactivation of the *RB1* gene in malignant brain tumours, we investigated the methylation status at the CpG island within the promoter region of the *RB1* gene. We also examined the essential promoter region and the protein-binding pocket domain (exons 20–24 and surrounding intronic sequences) for the presence of inactivating mutations.

## MATERIALS and Methods

### Tissue samples and DNA preparation

Fresh tumour tissues and blood samples were obtained from 136 patients with tumours of the nervous system, including: 42 (32 primary and 10 secondary) glioblastomas multiformes (GB), 21 WHO grade III AA, 22 WHO grade II oligodendrogliomas (O), 12 WHO grade III anaplastic oligodendrogliomas (AO), six WHO grade II–III mixed oligo-astrocytomas (OA), four WHO grade II ependymomas (E), three WHO grade III anaplastic ependymomas (AE), 11 medulloblastomas (MD), three primary central nervous system lymphomas (PCNSL), two neurofibrosarcomas (NFS), and 10 brain metastasis from solid tumours (MET). Tumours were diagnosed according to the WHO guidelines (Kleihues *et al*, 1993), and the tumour cell content was estimated by histologic examination to be approximately 75–90%. DNA was prepared from frozen tissues and blood samples using standard methods, as described (Rey *et al*, 1992).

### PCR/SSCP analysis and direct sequencing

The genomic DNA derived from tumour tissues and blood samples was used as template for PCR-based amplifications of the essential promoter region (encompassing nucleotides −300 to −174) and the protein-binding pocket domain (exons 20–24 and surrounding intronic sequences) of the *RB1* gene. We used the primers and PCR conditions as described by Simpson *et al*, (2000) (purchased from SIGMA ARK, St Louis, MO, USA). For SSCP analysis, the PCR products were loaded onto 6–12% nondenaturing polyacrylamide gels (with or without 10% glycerol), electrophoresed, and silver-stained. Samples displaying an altered PCR-SSCP pattern were reamplified by PCR with the same set of primers, and the PCR products were sequenced using an ABI PRISM BigDye Terminator Cycle Sequencing Ready Reaction Kit (Perkin-Elmer Applied Biosystems, Foster city, CA, USA) on the Applied Biosystem model 373A DNA sequencer. Each amplicon was sequenced bidirectionally.

### Bisulphite treatment of DNA and methylation-specific (MSP) PCR

Bisulphite modification of genomic DNA was performed as reported by Herman *et al*, (1996). Briefly, 2 *μ*g of genomic DNA was denatured with 2 mol l^−1^ NaOH (37°C for 10 min), followed by incubation with 3 mol l^−1^ sodium bisulphite (pH 5.0) at 50°C for 16 h in the dark. After treatment, DNA was purified using the DNA cleanup kit (Promega, Madison, WI, USA) as recommended by the manufacturer, incubated with 3 mol l^−1^ NaOH (room temperature for 5 min), precipitated with 10 mol l^−1^ ammonium acetate and 100% ethanol, washed with 70% ethanol and resuspended in 20 *μ*l distilled water. Primer sequences of *RB1* for the methylated and unmethylated reaction were as reported (Simpson *et al*, 2000). The PCR amplification was carried out in a thermal cycler using Amplitaq polymerase with denaturation at 95°C for 5 min, followed by 35 cycles of 95°C for 30 s, 55°C for 30 s, and 72°C for 30 s. The reaction was finished with a 7 min extension at 72°C. PCR products were electrophoresed on 3% agarose gels and visualised with ethidium bromide. To verify the identity of the PCR products, they were purified and sequenced as above for mutation analysis. In addition to tumours, two samples of non-neoplastic cerebral tissue obtained by autopsy were studied. As positive/negative controls for methylated alleles we used DNA (from lymphocytes of healthy volunteers), treated/not treated with Sss1 methyltransferase (New England Biolabs, Beverly, MA, USA) and then subjected to bisulphite treatment.

## RESULTS

### RB1 sequence analysis

*Promoter region*. No case showed mobility shifts by PCR-SSCP analysis of the *RB1* promoter region and, thus no inactivating mutations were found in any tumour examined.

**Figure 1:**
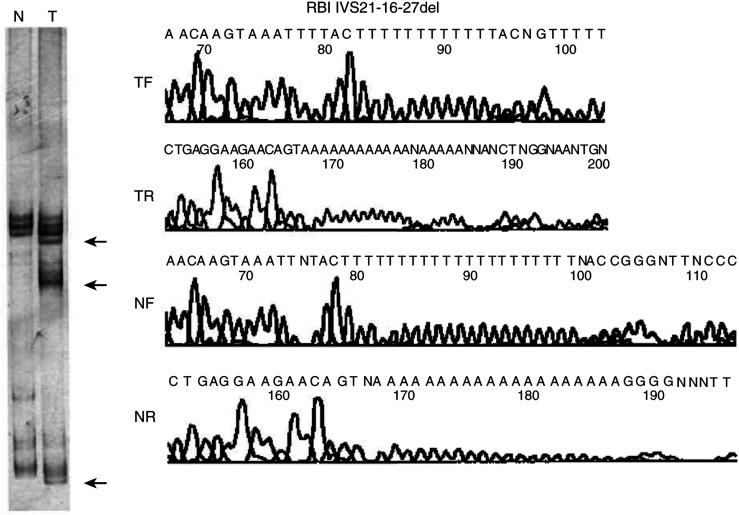
*RB1* intron 21 mutation in one glioblastoma. A deletion of 12 bp, at position –16 to –27 of intron 21, was identified by sequencing. To the left is shown the SSCP analysis corresponding to the constitutional (N) and tumoural (T) DNAs (mobility shifts are indicated by arrows). Forward and reverse sequences corresponding to the tumour and constitutional DNA show the nucleotide changes.

*Protein-binding pocket domain*. Only two tumours presented PCR-SSCP variations of the exons 20–24; they corresponded to one GB and one O displaying similar alterations. A mobility shift in the SSCP pattern of the PCR product for exon 22, which also includes the surrounding sequence for intron 21, was observed in both tumours (Figure 1

). Sequence analysis demonstrated a deletion involving 12 bp (in GB) and 8 bp (in O) at position −16 to −27, and −16 to −23 of intron 21 (IVS21-16–27del and IVS21-16–23del), respectively.

### RB1 promoter hypermethylation

*RB1* promoter hypermethylation was detected in 26 of the 136 cases studied (19%). Among glial tumours, aberrant methylation was evidenced in nine GB (five primary GB; four secondary GB), three AA, one OA, and one E. The remaining 12 cases corresponded to two MD, two PCNSL, two NFS, and six MET (two malignant melanoma, three ovarian carcinomas, and one breast carcinoma). Table 1

**Table 1:** Promoter hypermethylation of the *RB1* gene in nervous system tumours

		**No. of cases with**		
**Histology**	**No. of cases**	**Promoter hypermethylation**	**LOH at 13q^a^**	**Hypermethylation+LOH 13q**
Primary GBM	32	5 (15%)	5 (15%)	1
Secondary GBM	10	4 (40%)	3 (30%)	1
				
*GBM total*	*42*	*9 (21%)*	*8 (19%)*	*2*
				
AA	21	3 (14%)	4 (19%)	0
O	22	0	1 (5%)	—
AO	12	0	2 (17%)	—
OA	6	1 (16%)	0	—
E	4	1 (25%)	1 (25%)	0
AE	3	0	0	—
MD	11	2 (18%)	1 (9%)	0
PCNSL	3	2 (66%)	0	—
NFS	2	2 (100%)	0	—
MET	10	6 (60%)	5 (50%)	2
				
Total	136	26 (19%)	22 (16%)	4

**Figure 2:**
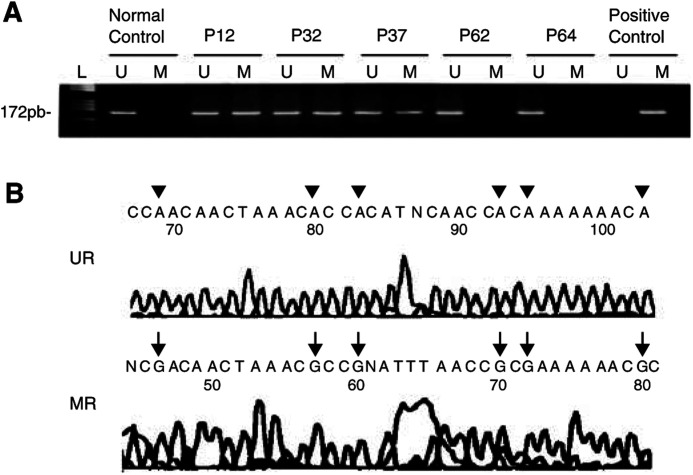
(**A**) Methylation-specific PCR of CpG island of the *RB1* promoter in glioblastomas (P12 and P32) and anaplastic astrocytoma (P37). Cases P62 and P64 (glioblastomas) showed only unmethylated alleles. Positive control for methylated DNA: normal DNA from lymphocytes treated with SssI methyltransferase; normal control: DNA from a non-neoplastic brain tissue. Negative control from untreated lymphocytes DNA is not shown (L: molecular weight marker). (**B**) Sequence (reverse of the coding strand) analysis of bisulphite-modified DNA from tumour P12 (MR) and from non-neoplastic brain tissue (UR). Tumour DNA shows methylated cytosines (G in the reverse sequencing marked by arrows) at the represented CpG sites, whereas all CpG cytosines are unmethylated in DNA from non-neoplastic brain tissue (A in the reverse sequencing marked by arrowheads).

GBM=glioblastoma; AA=WHO grade III anaplastic astrocytoma; O=WHO grade II oligodendroglioma; AO=WHO grade III anaplastic oligodendroglioma; OA=WHO grade II–III mixed oligo-astrocytoma; E=WHO grade II ependymoma; AE=WHO grade III Anaplastic ependymoma; MD=medulloblastoma; PCNLS=primary central nervous system lymphoma; NFS=Neurofibrosarcoma; MET=Brain metastasis from solid tumour.

a Results from Bello *et al*, (1994) and unpublished data.

shows a summary of the main findings. Methylated and unmethylated control DNAs displayed the expected fragment size of 172 bp, and sequencing of bisulphite-modified DNA of the tumours and control (non-neoplastic brain) PCR products demonstrated hypermethylation or normal sequences, respectively (Figure 2

).

## DISCUSSION

Abnormalities in expression of cell-cycle regulatory genes occur commonly in human malignancies. In addition to the childhood tumour retinoblastoma, *RB1* inactivation has also been demonstrated in a variety of tumours including sarcomas, lung, breast, and bladder carcinomas, as well as malignant gliomas (Cance *et al*, 1990; Xu *et al*, 1991; Logothetis *et al*, 1992; Trudel *et al*, 1992; Ichimura *et al*, 1996; Tsuzuki *et al*, 1996; Ueki *et al*, 1996). The inactivation of both *RB1* alleles in tumour cells was initially detected in association with LOH at the *RB1* locus on chromosome 13, and microdeletions or inactivating mutations in the retained allele would be responsible for complete inactivation of the gene (Ishikawa *et al*, 1991; Hogg *et al*, 1993; Xu *et al*, 1993; Gouyer *et al*, 1994; Henson *et al*, 1994; Ichimura *et al*, 1996). Loss of 13q arm has been found in about 0–30% of solid tumours, including nervous system neoplasms in which up to 45% of GB display loss at the *RB1* locus (Ishikawa *et al*, 1991; Hogg *et al*, 1993; Xu *et al*, 1993; Gouyer *et al*, 1994; Henson *et al*, 1994; Ichimura *et al*, 1996). We have previously screened this series of tumours for allelic constitution at chromosome 13 (Bello *et al*, 1994, and unpublished data) analysing two polymorphic loci (D13S4 and D13S63) as well as the *RB1* locus with a cDNA probe used for densitometric analysis on *Taq*I/*Msp*I Southern blots. We detected 16% of samples characterised by 13q losses, primarily involving GB (eight cases), AA (four samples), O (one case), AO (two cases), E (one tumour), MD (one case), and MET (five tumours). We did not detect any homozygous deletion in our series.

Initial studies suggested that allelic losses of chromosome 13q arm were accompanied by inactivating mutations in the gene (Ishikawa *et al*, 1991; Hogg *et al*, 1993; Xu *et al*, 1993; Gouyer *et al*, 1994; Henson *et al*, 1994; Ichimura *et al*, 1996). Nonetheless, the finding of tumours displaying LOH at the *RB1* locus without any abnormality in the remaining *RB1* allele, but with the absence of pRB expression (Borg *et al*, 1992; Cooney *et al*, 1996; Ueki *et al*, 1996; Burns *et al*, 1998; Simpson *et al*, 1999), supports the existence of mutations in regions such as promoter or introns, which are frequently not explored or escape SSCP studies. Moreover, *RB1* gene function loss has been found involving mutations in both alleles with retention of heterozygosity at the *RB1* locus, as described in bladder carcinomas (Ishikawa *et al*, 1991). Mutations or microdeletions at exons 20–24 have primarily been associated with an absence of pRB or reduced *RB1* transcript (Yandell *et al*, 1989; Hensel *et al*, 1990; Mori *et al*, 1990; Bookstein *et al*, 1990b). We found no alterations at the essential promoter region, and only two tumours (diagnosed as GB and O) displayed sequence anomalies at the protein-binding pocket domain (exons 20–24) we screened by SSCP; both tumours retained two *RB1* alleles. Henson *et al*, (1994) studied all 27 exons and flanking intronic regions of the *RB1* in a series of 85 astrocytic tumours and glioblastomas. Three of the four mutations they identified were located in this region, involving exon 24 (two instances) and intron 24 (one case). Tsuzuki *et al*, (1996) analysed 23 brain tumour specimens with astrocytic differentiation. Sequence variations were identified in three instances, one of them involving codon 754 in exon 22: GTA-to-GGA transversion, resulting in a Val-to-Gly substitution. Ichimura *et al*, (1996) performed *RB1* mutation analysis of 195 astrocytic gliomas. In addition to three homozygous deletions, *RB1* gene mutations were detected in 12% of samples; one of these was located at exons 20–24. This consisted of a duplication–insertion of 24 bases in exon 21 that led to an in-frame insertion of eight amino acids. All these reports thus show little mutational involvement of the *RB1* essential promoter region, and a low frequency of alterations at the protein-binding domain (exons 21–24) in astrocytic neoplasms.

The discrepancies observed among the rates of LOH at 13q (30% of cases), *RB1* inactivating mutations (5–12% of cases), homozygous *RB1* deletions (3% of tumours), and loss of *RB1* expression detected by immunohistochemical analysis (up to 27% of samples), strongly suggest that other molecular mechanisms may participate in the inactivation of this gene; moreover, loss of *RB1* expression does not always correlate with LOH at the *RB1* locus (Henson *et al*, 1994; Ichimura *et al*, 1996; Nakamura *et al*, 1996; Ueki *et al*, 1996; Burns *et al*, 1998).

Methylation of gene regulatory elements has been recognised as an important mechanism participating in gene inactivation (Esteller and Herman, 2002). Initial studies using methylation-sensitive restriction enzyme digest techniques demonstrated that methylation at the promoter region and exon 1 of *RB1* gene is associated with reduced levels of *RB1* transcript (Greger *et al*, 1994). At present, hypermethylation is accepted as a mechanism of *RB1* gene inactivation and, in pituitary adenomas, loss of pRB expression has been found to be associated with methylation of the CpG island within the *RB1* promoter region together with deletion within the protein-binding pocket domain (Simpson *et al*, 2000).

Our study showed an overall *RB1* gene methylation rate incidence of 19%, with the highest frequency detected in the MET group (60%). This finding would concur with the high grade of malignancy characteristic of metastatic tumours. They would accumulate several genetic alterations and probably epigenetic changes involving inactivation of regulatory elements of key genes participating in cell-cycle regulation and cell growth control (Seike *et al*, 2000). A high methylation rate was also observed in NFS (two cases of two analysed) and PCNSL (two cases of three studied). Although the low number of samples available for analysis makes it difficult to draw firm conclusions, the data suggest that epigenetic inactivation of *RB1* gene in parallel to methylation is a frequent mechanism that contributes to tumour development or progression in these neoplasms. With regard to malignant glial tumours, we found significant *RB1* methylation rates in the group of GB (21% of samples). These figures are slightly lower than those reported by Nakamura *et al*, (2001), who identified promoter hypermethylation in the *RB1* gene in one-quarter (14 of 56 cases) of the GB they studied. These authors also demonstrated that the majority of GB with loss of *RB1* expression had *RB1* promoter hypermethylation, whereas the majority of tumours with *RB1* expression had normal *RB1* gene status. We might thus consider that most cases with hypermethylated *RB1* promoter in our series most probably would show loss of pRB expression, although we had no possibility to perform pRB expression studies in our series of tumours to demonstrate this. Our findings therefore support the hypothesis that promoter hypermethylation is an epigenetic mechanism frequently involved in the loss of *RB1* function in GB. In agreement with the data provided by Nakamura *et al*, (2001), we detected *RB1* methylation more frequently in secondary than in primary GB (40 *vs* 15% of cases, respectively). We found hypermethylation in three of the 21 (14%) AA we studied, whereas no case of the 10 AA tumours gave positive results in the series of Nakamura *et al*, (2001). One E and one OA in our series were also characterised by *RB1* promoter hypermethylation, whereas no O nor AO displayed this alteration. No previous data are available on *RB1* methylation in E, and our findings in tumours with a major oligodendroglial component contrast with those reported by Dong *et al*, (2001), who detected methylation in 34% (14 of 26) cases studied. On the other hand, Watanabe *et al*, (2001) identified this anomaly in three of 48 tumours, suggesting that promoter alteration of *RB1* is rare in oligodendrogliomas, and demonstrated that inactivation of the RB1-mediated G1->S cell-cycle transition pathway is more frequently caused by *CDK4* amplification or *p16*^*INK4A*^*/p15*^*INK4B*^ inactivation.

Loss of *RB1* expression is associated with a higher grade of malignancy and appears to be a prognostic factor in several human neoplasms (Cryns *et al*, 1994; Nakamura *et al*, 2001). If our finding on *RB1* methylation in three AA is confirmed in a larger series, it may be representative of an AA subgroup with a more aggressive biological behaviour.

## References

[bib1] Baylin SB, Herman JG, Graff JR, Vertino PM, Issa JP (1998) Alterations in DNA methylation: a fundamental aspect of neoplasia. Adv Cancer Res 72: 141–196 9338076

[bib2] Bello MJ, de Campos JM, Kusak ME, Vaquero J, Sarasa JL, Pestaña A, Rey JA (1994) Molecular analysis of genomic abnormalities in human gliomas. Cancer Genet Cytogenet 73: 122–129 8174086 10.1016/0165-4608(94)90195-3

[bib3] Bookstein R, Rio P, Madrepeira SA, Homg F, Allred C, Grizzle WE, Lee WH (1990a) Promoter deletion and loss of retinoblastoma gene expression in human prostate carcinoma. Proc Natl Acad Sci USA 87: 7762–7766 2217208 10.1073/pnas.87.19.7762 PMC54828

[bib4] Bookstein R, Shew J-Y, Chen P-L, Scully P, Lee W-H (1990b) Supression of tumourigenicity of human prostate carcinoma cells by replacing a mutated *RB1* gene. Science 247: 712–715 2300823 10.1126/science.2300823

[bib5] Borg A, Zhang QX, Alm P, Olsson H, Sellberg G (1992) The retinoblastoma gene in breast cancer: allelic loss is not correlated with loss of gene protein expression. Cancer Res 52: 2991–2994 1581913

[bib6] Buchkovich K, Duffy LA, Harlow E (1989) The retinoblastoma gene is phosphorylated during specific phases of the cell cycle. Cell 58: 1097–1105 2673543 10.1016/0092-8674(89)90508-4

[bib7] Burns KL, Ueki K, Jhung SL, Koh J, Louis DN (1998) Molecular genetic correlates of p16, cdk4, and pRb immunohistochemistry in glioblastomas. J Neuropathol Exp Neurol 57: 122–130 9600204 10.1097/00005072-199802000-00003

[bib8] Cance WG, Brennan MF, Dudas ME, Huang CM, Cordon-Cardo C (1990) Altered expression of the retinoblastoma gene product in human sarcomas. N Engl J Med 323: 1457–1462 2233918 10.1056/NEJM199011223232105

[bib9] Cooney KA, Wetzel JC, Merajver SD, Macoska JA, Singleton TP, Wojno KJ (1996) Distinct regions of allelic loss on 13q in prostate cancer. Cancer Res 56: 1142–1145 8640774

[bib10] Cryns VL, Thor A, Xu HJ, Hu SX, Wierman ME, Vickery AL, Benedict WF, Arnold A (1994) Loss of retinoblastoma tumour-suppressor gene in parathyroid carcinoma. N Engl J Med 330: 757–761 7906387 10.1056/NEJM199403173301105

[bib11] Delgado S, Gomez M, Bird A, Antequera F (1998) Initiation of DNA replication at CpG islands in mammalian chromosomes. EMBO J 17: 2426–2435 9545253 10.1093/emboj/17.8.2426 PMC1170585

[bib12] Dong S-M, Pang JC-S, Poon W-S, Hu J, Yo K-F, Chang AR, Ng H-K (2001) Concurrent hypermethylation of multiple genes is associated with grade of oligodendroglial tumours. J Neuropathol Exp Neurol 60: 808–816 11487055 10.1093/jnen/60.8.808

[bib13] Esteller M, Herman JG (2002) Cancer as an epigenetic disease: DNA methylation and chromatin alterations in human tumours. J Pathol 196: 1–7 11748635 10.1002/path.1024

[bib14] Friend SH, Bernards R, Rogelji S, Weinberg RA, Rapaport JM, Alberts DM, Dryja TP (1986) A human DNA segment with properties of the gene that predisposes to retinoblastoma and osteosarcoma. Nature 323: 643–646 2877398 10.1038/323643a0

[bib15] Gouyer V, Gazzeri S, Brambilla E, Bolon I, Moro D, Perron P, Benabid AL, Brambilla C (1994) Loss of heterozygosity at the RB locus correlates with loss of RB protein in primary malignant neuroendocrine lung carcinoma. Int J Cancer 58: 818–824 7927874 10.1002/ijc.2910580612

[bib16] Greger V, Debus N, Lohmann D, Hopping W, Passarge E, Horsthemke B (1994) Frequency and parental origin of hypermethylated *RB1* alleles in retinoblastoma. Hum Genet 94: 491–496 7959682 10.1007/BF00211013

[bib17] Hensel CH, Hsieh C-L, Gazdar AF, Johnson BE, Sakaguchi AY, Naylor SL, Lee W-H, Lee EY-HP (1990) Altered structure and expression of the human retinoblastoma susceptibility gene in small cell lung cancer. Cancer Res 50: 3067–3072 2159370

[bib18] Henson JW, Schnitker BL, Correa KM, von Deimling A, Fassbender F, Xu H-J, Benedict WF, Yandell DW, Louis DN (1994) The retinoblastoma gene is involved in malignant progression of astrocytomas. Ann Neurol 36: 714–721 7979217 10.1002/ana.410360505

[bib19] Herman JG, Graff JR, Myohanen S, Nelkin BD, Baylin SB (1996) Methylation-specific PCR: a novel PCR assay for methylation status of CpG islands. Proc Natl Acad Sci USA 93: 9821–9826 8790415 10.1073/pnas.93.18.9821 PMC38513

[bib20] Hogg A, Bia B, Onadim Z, Cowell K (1993) Molecular mechanisms of oncogenetic mutations in tumours from patients with bilateral and unilateral retinoblastoma. Proc Natl Acad Sci USA 90: 7351–7355 8346255 10.1073/pnas.90.15.7351 PMC47135

[bib21] Ichimura K, Schmidt EE, Goike HM, Collins VP (1996) Human glioblastomas with no alterations of the *CDKN2A (p16^INK4A^, MTS1)* and *CDK4* genes have frequent mutations of the retinoblastoma gene. Oncogene 13: 1065–1072 8806696

[bib22] Ishikawa J, Xu H-J, Yandell DW, Maeda S, Kamidono S, Benedict WF, Takahashi R (1991) Inactivation of the retinoblastoma gene in human bladder and renal cell carcinomas. Cancer Res 51: 5736–5743 1913692

[bib23] Jones PA (1996) DNA methylation errors and cancer. Cancer Res 56: q2463–2467 8653676

[bib24] Jones PA, Laird PW (1999) Cancer epigenetic comes of age. Nat Genet 21: 163–167 9988266 10.1038/5947

[bib25] Kleihues P, Burger PC, Scheitauer BW (1993) Histological typing of tumours of the nervous system. WHO International Histological Classification of Tumours, 2nd edn. Berlin: Springer-Verlag

[bib26] Lee WH, Shew JY, Hong F, Sery T, Donoso LA, Young LJ, Bookstein R, Lee EYHP (1987) The retinoblastoma susceptibility gene product is a nuclear phosphoprotein associated with DNA binding activity. Nature 329: 642–645 3657987 10.1038/329642a0

[bib27] Logothetis CJ, Xu HJ, Ro JY, Xu SX, Sahin A, Ordonez N, Benedict WF (1992) Altered expression of retinoblastoma protein and known prognostic variables in locally advanced bladder cancer. J Natl Cancer Inst 84: 1256–1261 1640485 10.1093/jnci/84.16.1256

[bib28] Medema RH, Herrera RE, Lam F, Harlow RA (1995) Growth suppression by p16^ink4^ requires functional retinoblastoma protein. Proc Natl Acad Sci USA 92: 6289–6293 7603984 10.1073/pnas.92.14.6289 PMC41503

[bib29] Mori N, Yokota J, Akiyama T, Sameshima Y, Okamoto A, Mizoguchi H, Toyoshima K, Sugimura T, Terada M (1990) Variable mutation of the RB gene in small-cell lung carcinoma. Oncogene 11: 1713–1717 2176283

[bib30] Nakamura M, Konishi N, Hiasa Y, Tsunoda S, Fukushima Y, Tsuzuki T, Takemura K, Aoki H, Kobitsu K, Sakaki Y (1996) Immunohistochemical detection of *CDKN2*, retinoblastoma and *p53* gene products in primary astrocytic tumours. Int J Oncol 8: 889–893 21544442 10.3892/ijo.8.5.889

[bib31] Nakamura M, Yonekawa Y, Kleihues P, Ohgaki H (2001) Promoter hypermethylation of the *RB1* gene in glioblastomas. Lab Invest 81: 77–82 11204276 10.1038/labinvest.3780213

[bib32] Ohtani-Fujita N, Fujita T, Aoike A, Osifchin NE, Robbins PD, Sakai T (1993) CpG methylation inactivates the promoter activity of the human retinoblastoma tumour-suppressor gene. Oncogene 8: 1063–1067 8455933

[bib33] Rey JA, Bello MJ, Jimenez-Lara A, Vaquero J, Kusak ME, de Campos JM, Sarasa JL, Pestaña A (1992) Loss of heterozygosity for distal markers on 22q in human gliomas. Int J Cancer 51: 703–706 1351884 10.1002/ijc.2910510507

[bib34] Sakai T, Ohtami N, McGee TL, Robbins PD, Dryja TP (1991) Oncogenic germ-line mutations in SpI and ATF sites in the human retinoblastoma gene. Nature 353: 83–86 1881452 10.1038/353083a0

[bib35] Seike M, Gemma A, Hosoya Y, Hemmi S, Taniguchi Y, Fukuda Y, Yamanaka N, Kudoh S (2000) Increase in the frequency of *p16^INK4^* gene inactivation by hypermethylaton in lung cancer during the process of metastasis and its relation to the status of *p53*. Clin Cancer Res 6: 4307–4313 11106248

[bib36] Simpson DJ, Magnay J, Bicknell JE, Barkan AL, McNicol AM, Clayton RN, Farrell WE (1999) Chromosome 13q deletion mapping in pituitary tumours: infrequent loss of the retinoblastoma susceptibility gene (RB1) locus despite loss of RB1 protein product in somatotrophinomas. Cancer Res 59: 2703–2709 10197629

[bib37] Simpson DJ, Hibberts NA, McNicol AM, Clayton RN, Farrell WE (2000) Loss of pRb expression in pituitary adenomas is associated with methylation of the *RB1* CpG island. Cancer Res 60: 1211–1216 10728677

[bib38] Stirzaker C, Millar S, Paul CL, Warnecke PM, Harrison J, Vincent PC, Frommer M, Clark SJ (1997) Extensive DNA methylation spanning the Rb promoter in retinoblastoma tumours. Cancer Res 57: 2229–2237 9187126

[bib39] Trudel M, Mulligna L, Cavenee W, Margolese R, Cote J, Gariepy G (1992) Retinoblastoma and *p53* gene product expression in breast carcinoma: immunohistochemical analysis and clinicopathologic correlation. Hum Pathol 23: 1388–1394 1468776 10.1016/0046-8177(92)90059-c

[bib40] Tsuzuki T, Tsunoda S, Sakaki T, Konishi N, Hiasa Y, Nakamura M (1996) Alterations of retinoblastoma, *p53*, *p16(CDKN2)*, and *p15* genes in human astrocytomas. Cancer 78: 287–293 8674005 10.1002/(SICI)1097-0142(19960715)78:2<287::AID-CNCR15>3.0.CO;2-S

[bib41] Ueki K, Ono Y, Henson JW, Efird JT, von Deimling A, Louis DN (1996) *CDKN2/p16* or *RB* alterations occur in the majority of glioblastomas and are inversely correlated. Cancer Res 56: 150–153 8548755

[bib42] Watanabe T, Yokoo H, Yokoo M, Yonekawa Y, Kleihues P, Ohgaki H (2001) Concurrent inactivation of RB1 and TP53 pathways in anaplastic oligodendrogliomas. J Neuropathol Exp Neurol 60: 1181–1189 11764090 10.1093/jnen/60.12.1181

[bib43] Xu HJ, Xu SX, Cagle PT, Moore GE, Benedict WF (1991) Absence of retinoblastoma protein expression in primary non-small cell lung carcinomas. Cancer Res 51: 2735–2739 1850661

[bib44] Xu HJ, Cairns P, Hu SX, Knowles MA, Benedict WF (1993) Loss of RB protein expression in primary bladder cancer correlates with loss of heterozygosity at the RB locus and tumour progression. Int J Cancer 53: 781–784 8449603 10.1002/ijc.2910530513

[bib45] Yandell DW, Campbell TA, Dayton SH, Petersen R, Walton D, Little JB, McConkie-Rosell A, Buckley EG, Dryja TP (1989) Oncogeneic point mutations in the human retinoblastoma gene: their application to genetic counselling. N Engl J Med 21: 1689–1695 10.1056/NEJM198912213212501 2594029

